# Prognostic value of protein inhibitor of activated STAT3 in breast cancer patients receiving hormone therapy

**DOI:** 10.1186/s12885-016-2063-1

**Published:** 2016-01-14

**Authors:** Sheau-Fang Yang, Ming-Feng Hou, Fang-Ming Chen, Fu Ou-Yang, Yang-Chang Wu, Chee-Yin Chai, Yao-Tsung Yeh

**Affiliations:** Department of Pathology, Kaohsiung Municipal Ta-Tung Hospital, No. 68, Zhonghua 3rd Rd, Qianjin Dist, Kaohsiung, 801 Taiwan R O C; Department of Pathology, Faculty of Medicine, College of Medicine, Kaohsiung Medical University, No.100, Shiquan 1st Rd, Sanmin Dist, Kaohsiung, 807 Taiwan R O C; Department of Surgery, Kaohsiung Municipal Ta-Tung Hospital, No. 68, Zhonghua 3rd Rd, Qianjin Dist, Kaohsiung, 801 Taiwan R O C; Department of Surgery, Faculty of Medicine, College of Medicine, Kaohsiung Medical University, No. 100, Shiquan 1st Rd, Sanmin Dist, Kaohsiung, 807 Taiwan R O C; Department of Laboratory, Kaohsiung Municipal Ta-Tung Hospital, No. 68, Zhonghua 3rd Rd, Qianjin Dist, Kaohsiung, 801 Taiwan R O C; School of Pharmacy, College of Pharmacy, China Medical University, No. 91, Hsueh-Shih Road, Taichung, Taiwan 40402 R O C; Chinese Medicine Research and Development Center, China Medical University Hospital, No. 2, Yude Road, Taichung, 40447 Taiwan R O C; Department of Medical Laboratory Sciences and Biotechnology, Fooyin University, No.151, Jinxue Rd, Daliao Dist, Kaohsiung, 831 Taiwan R O C

**Keywords:** Breast cancer, Protein inhibitor of activated STAT3, Estrogen receptor, Cyclin D1, Tamoxifen

## Abstract

**Background:**

Deregulated signal transducer and activator of transcription 3 (STAT3) signaling has been well documented in certain cancers. Alterations in specific negative regulators, such as protein inhibitor of activated STAT3 (PIAS3), may contribute to cancer development.

**Methods:**

The expression of total PIAS3 was determined in 100 paired cancerous and non-cancerous breast tissues by immunoblotting and was statistically analyzed along with the clinicopathological characteristics and overall survival of the patients. XTT, immunoblotting, and chromatin immunoprecipitation (Chip) were used to examine the biological effect of PIAS3 in breast cancer cells.

**Results:**

Hormone therapy failed to improve the overall survival in patients presenting with increased PIAS3 expression. Ectopic PIAS3 overexpression increased the proliferation and expression of cyclin D1 in estrogen receptor (ER)-positive MCF-7 and T47D cells, but decreased those in ER-negative MDA-MB-231 and SKBR3 cells. Furthermore, PIAS3 overexpression attenuated cytotoxicity of tamoxifen and increased proliferation and cyclin D1 expression in MCF-7 cells. PIAS3 also decreased the binding of itself on the *cyclin D1* promoter and this decreased binding was not affected by tamoxifen.

**Conclusion:**

PIAS3 may serve as a biomarker for predicting hormone therapy stratification, although it is limited to those breast cancer patients receiving hormone therapy

**Electronic supplementary material:**

The online version of this article (doi:10.1186/s12885-016-2063-1) contains supplementary material, which is available to authorized users.

## Background

Deregulated Janus-activated kinase (JAK)/signal transducer and activator of transcription (STAT) signaling has been closely associated with various human diseases. In this pathway, STAT3 is the most recognized oncogene and frequently is activated in human cancers [1]. Aberrations in a specific STAT3 regulator such as the protein inhibitor of activated STAT3 (PIAS3) may also contribute to cancer development [2]. The PIAS3 transcript is rarely detected in certain cancers such as mesotheliomas [3] and lymphoma cells, and loss of PIAS3 expression is responsible for constitutive STAT3 activation [4]. In addition, ectopic expression of PIAS3 can suppress prostate cancer cells through inducing apoptosis in vivo and in vitro [5].

It is well documented that PIAS3 can inhibit the DNA-binding activity of STAT3 and subsequently repress the transcription activity of STAT3 [6]. Nevertheless, it is important to note that p-tyr705-STAT3 nuclear expression positive breast cancer has a significantly improved short-term (5-year) survival and long-term (20-year) survival [7]. In addition, positive p-tyr705-STAT3 nuclear expression has been shown to be an independent prognostic marker of better overall survival in node-negative breast cancer using multivariate analyses. Other studies have suggested that tyrosine 705 phosphorylation of STAT3 is a marker of good prognosis, at least in breast cancer [8, 9]. Thus, inhibition of STAT3 activity may specifically promote further development of breast cancer under certain conditions. Interestingly, silencing the expression of the *PIAS3* gene significantly downregulates the expression of estrogen receptor (ER) and its downstream targets in MCF-7 cells [10]. In addition, PIAS3 acts as a SUMO-1 E3 ligase for ERα sumoylation [11]. ERα sumoylation is strictly ligand (e.g., estrogen)-dependent in the presence of PIAS3 and concomitant expression of PIAS3 with ERα strongly activates estrogen-dependent transcription [11], suggesting that PIAS3 may facilitate ER signaling. It is controversial whether estrogen and ER are able to inhibit STAT3 signaling [12]. Estrogen induces PIAS3 expression and increases the physical association between PIAS3 and STAT3 to block DNA binding and transactivation of STAT3, suggesting that PIAS3 may serve as a co-regulator that modulates crosstalk between ER and STAT3 [12]. These results suggest that interplay among ER, STAT3, and PIAS3 may have a role in estrogen-dependent malignancies such as breast cancer.

Constitutively activated STAT3 has been associated with various malignancies [13–15]. Deregulation of its negative regulatory system may play a role in breast cancer. In the present study, we explored whether the expression of total PIAS3 protein was aberrantly expressed and whether these alterations contributed to breast cancer progression. We used immunoblotting to analyze the levels of total PIAS3 in paired cancerous and adjacent noncancerous breast tissues. We found that increased total PIAS3 protein may predict poor prognosis. Our results suggest that PIAS3 may play a role in breast cancer, along with ER.

## Methods

### Tissue sample preparations

A total of 100 patients with pathologically confirmed breast cancer were included in this study. Paired cancerous and noncancerous breast tissues were obtained from the 100 patients who had undergone surgical treatment at the Department of Surgery, Kaohsiung Medical University Hospital (KMUH) from 2001 to 2009. None of the patients had undergone radiotherapy or chemotherapy before surgery. The Institutional Review Board of Kaoshiung Medical University Hospital approved the study (KMUH-IRB-970222), and a written informed consent was obtained from each patient. Specimens from patients were frozen immediately in liquid N_2_, stored at −80 °C and/or were routinely fixed in 10 % buffered formalin and embedded in paraffin wax until further analysis. Chemotherapy included six cycles of fluorouracil, epirubicin, and cyclophosphamide or six cycles of docetaxel, epirubicin, and cyclophosphamide. In accordance with the National Comprehensive Cancer Network guidelines, chemotherapy was mainly administered to lymph node-negative patients, while hormonal therapy [usually tamoxifen (Tam) and anastrozole] was mainly administered to ER-positive patients.

### Western blot analysis

Frozen specimens were ground in liquid N_2_ and dissolved in Triton X-100 lysis buffer. The cells were also dissolved in the same lysis buffer. The extracts were centrifuged at 13,200 rpm for 20 min at 4 °C. The protein concentration in the supernatant was determined by Bradford assay using the Bio-Rad protein assay kit (Bio-Rad, Hercules, CA, USA). Equal amounts of total protein (~150 μg) were separated on 8 ~ 12 % sodium dodecyl sulfate (SDS)-polyacrylamide gel electrophoresis gels, transferred to nitrocellulose membranes using a Semi-Dry Transfer Cell (Bio-Rad), hybridized with primary antibody for 2 h at room temperature (RT), identified with a secondary antibody for 1 h at RT, and exposed to Kodak film. Rabbit polyclonal anti-PIAS3 (H-169), mouse monoclonal anti-p-tyr705-STAT3 (B-7), anti-STAT3 (F-2), anti-ERα (D-12), and anti-cyclin D1 (HD-11) were purchased from Santa Cruz Biotechnology (Dallas, TX, USA).

### Cell proliferation assay

MCF-7, T47D, MDA-MB-231, and SKBR3 breast cancer cells were grown in six-well plates. The cells were at 60 % confluence when they were transfected with the PIAS3 expression plasmid using Lipofectamine™ 2000 reagent (Invitrogen, Carlsbad, CA, USA), and treated with or without Tam (10^−8^M) (Sigma-Aldrich, St. Louis, MO, USA). The cells were grown in Dulbecco’s modified Eagle’s medium supplemented with 10 % fetal bovine serum for 24 h and were harvested for XTT analysis.

### Chromatin immunoprecipitation

The cells were fixed in 1 % formaldehyde for 10 min, washed, and lysed in 500 μl cell lysis buffer (5 mM HEPES, pH 8.0; 85 mM KCl; and 0.5 % NP-40) at 4 °C for 10 min. The nuclei were released using a Dounce homogenizer and lysed in 100 ~ 200 μl nuclei lysis buffer (50 mM Tris–HCl, pH 8.0; 10 mM EDTA; and 1 % SDS). The lysate was sonicated on ice, and the supernatant was diluted 10-fold with dilution buffer (0.01 % SDS; 1.1 % Triton X-100; 1.2 mM EDTA; 16.7 mM Tris–HCl, pH 6.8; 167 mM NaCl). Anti-PIAS3 antibody (1 μg, Santa Cruz Biotechnology) was added to 0.5 ~ 1.0 ml lysate and the sample was incubated at 4 °C overnight. Immunocomplexes were then pulled down using protein G-conjugated magnetic Dynabeads (Dynal Biotech, Carlsbad, CA, USA). The beads were washed three times with wash buffer (0.1 M sodium phosphate buffer, pH 6.8; 0.1 % Tween-20), and the bound protein was eluted twice with 30 μl 0.1 M citrate (pH 3.0). Afterwards, 240 μl extraction buffer (0.1 % SDS, 50 mM NaHCO_3_, 5 μl of 10 mg/ml RNase, 18 μl of 5 M NaCl) was added to the pooled eluent and incubated at 65 °C overnight. The reverted DNA was purified with a Miniprep spin column (Qiagen, Valencia, CA, USA) and eluted in 50 μl of 10 mM Tris–HCl (pH 8.0). Polymerase chain reaction (PCR) was performed with *Taq* DNA polymerase (Bioman, New Delhi, India) under the following conditions: preheating at 95 °C for 2 min, followed by 35 cycles at 95 °C for 30 s, 56 °C for 30 s, and 72 °C for 45 s; with a final extension at 72 °C for 8 min. The PCR products were visualized and analyzed by electrophoresis on 2 % agarose gels containing ethidium bromide. The primers for the *cyclin D1* gene promoter region were as follows: 5′-CGAACACCTATCGATTTTGCTAA-′3 and 5′-TTGACCAGTCGGTCCTTGCGG-3. Representative experiments from three independent experiments are shown in Fig. 3d.

### Statistical analysis

**Fig. 1:**
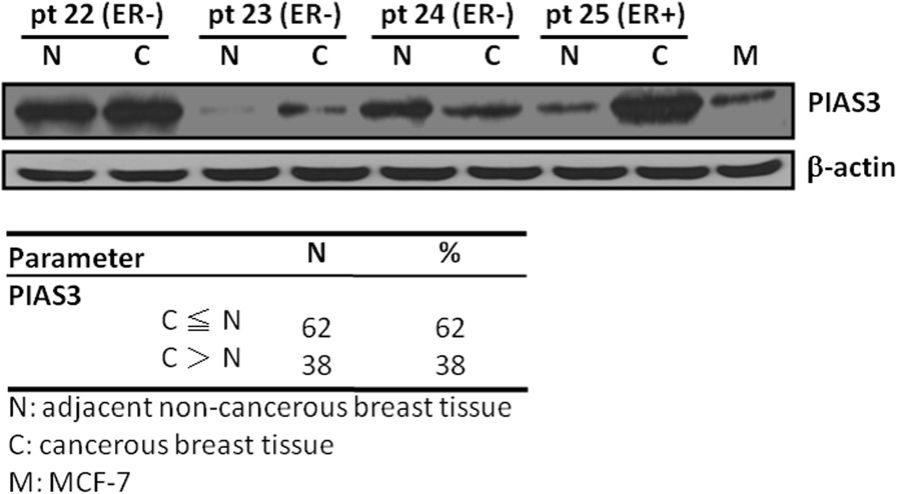
Protein inhibitor of activated signal transducer and activator- of transcription 3 (PIAS3) was detected in clinical breast cancer specimens. Some examples of the immunoblotting results. Total PIAS3 was determined by immunobotting in the breast cancer tissues (C) compared with the adjacent noncancerous tissues (N). The PIAS3 expression levels were normalized to the levels of the corresponding β-actin protein. Image J software was used to compare the expression levels of the total PIAS3 in the breast cancer tissues and the adjacent noncancerous tissues from the same patient after normalization with β-actin. C > N was defined as the ratio of total PIAS3 to the respective β-actin that was at least 50 % higher in the cancerous tissue than in the paired noncancerous tissue. Similarly, C < N was defined as the ratio of the total PIAS3 to the respective β-actin in the noncancerous tissue that was at least 50 % higher than that in the paired cancerous tissue. C = N was defined as less than 50 % difference in the ratio of the total PIAS3 to the respective β-actin between the two paired tissues. M: MCF-7 cell line

Statistical analyses were performed using the SPSS 18.0 statistical package (SPSS, Inc., Chicago, IL, USA) for PC. PIAS3 expression levels were normalized to the levels of the corresponding β-actin protein. Image J software was used to compare the expression levels of total PIAS3 in breast cancer tissues and the adjacent noncancerous tissues from the same patient after normalization with β-actin. C > N was defined as the ratio of total PIAS3 to the respective β-actin that was at least 50 % higher in the cancerous tissue than that in the paired noncancerous tissue. Similarly, C < N was defined as the ratio of total PIAS3 protein to the respective β-actin in noncancerous tissue that was at least 50 % higher than that in the paired cancerous tissue. C = N was defined as less than 50 % difference in the ratio of total PIAS3 protein to the respective β-actin between the two paired tissues. In the present study, the C = N (23 %) and C < N (39 %) groups were categorized as one C ≦ N group (Fig. 1). Groups of patients with different PIAS3 expression levels were correlated with ER status, progesterone receptor (PR) status, human epidermal growth factor receptor 2 (Her2) status, tumor stage, tumor grade, lymph node metastasis, and recurrence which were available in the patients’ records, using Spearman’s rho coefficient analysis and the chi-square test. Survival curves were calculated using the Kaplan–Meier (K–M) method. Significance was determined using the log rank test and a *P* value of ≤ 0.05 was considered significant [16–18].

## Results

**Fig. 2:**
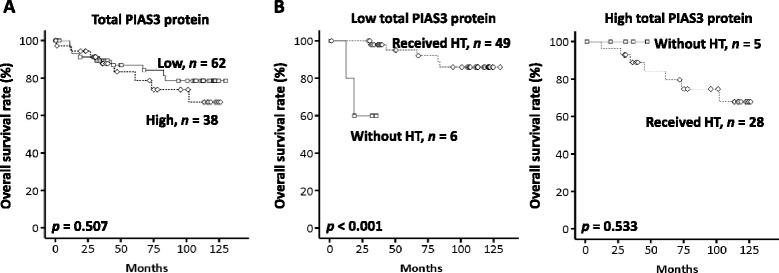
Kaplan-Meier analysis for overall survival of patients with breast cancer. **a** Survival curves of patients with high or low expression levels of total protein inhibitor of the PIAS3 in paired cancerous and noncancerous breast tissues. **b** Survival curves of patients with different expression levels of total PIAS3 after hormone therapy or no hormone therapy

Deregulated STAT3 signaling has been associated with breast cancer [9, 19] and thus, alterations in PIAS3 may also play a role in breast cancer. To determine the prognostic significance of PIAS3 in patients with breast cancer, we first used immunoblotting to analyze the expression patterns in paired cancerous and adjacent noncancerous breast tissues from the same patient. Total PIAS3 protein decreased in 62 % of the 100 breast cancer tissues compared with that in the adjacent noncancerous tissues (Fig. 1). Nevertheless, no significant correlation was observed between total PIAS3 protein expression levels and the clinicopatholgical characteristics as determined by *X*^*2*^ analysis (Table 1). Patients with decreased PIAS3 expression in tumor tissues tended to have advanced tumor stage and positive node metastasis (Table 1). The Kaplan–Meier (K-M) survival curves revealed that the PIAS3 expression levels are not directly associated with overall survival (Fig. 2a). Nevertheless, hormone therapy only improved overall survival in patients who presented with decreased PIAS3 expression levels (Fig. 2b). Radiotherapy or chemotherapy did not result in any improvement in the overall survival of patients with either high or low PIAS3 expression (data not shown).

**Table 1:** Correlation of total PIAS3 with clinicopathological characteristics in breast cancer

Characteristics	n (%)	PIAS3		*P* value^a^
		C ≦ N	C > N	
Stage				0.064
I	30 (30.0)	14	16	
II	44 (44.0)	28	16	
III	26 (26.0)	20	6	
Estrogen receptor status				0.852
Negative	38 (38.0)	24	14	
Positive	62 (62.0)	38	24	
Progesterone receptor status				0.843
Negative	54 (54.0)	33	21	
Positive	46 (46.0)	29	17	
Her2/Neu status^b^				0.193
Negative	34 (35.1)	24	10	
Positive	63 (64.9)	36	27	
Lymph node metastasis status				0.071
Absent	49 (49.0)	26	23	
Present	51 (51.0)	36	15	

N, adjacent non-cancerous breast tissue; C, cancerous breast tissue; ^a^, *X* ^*2*^ *test;* ^b^, not determined in a small subset of the cases

**Fig. 3:**
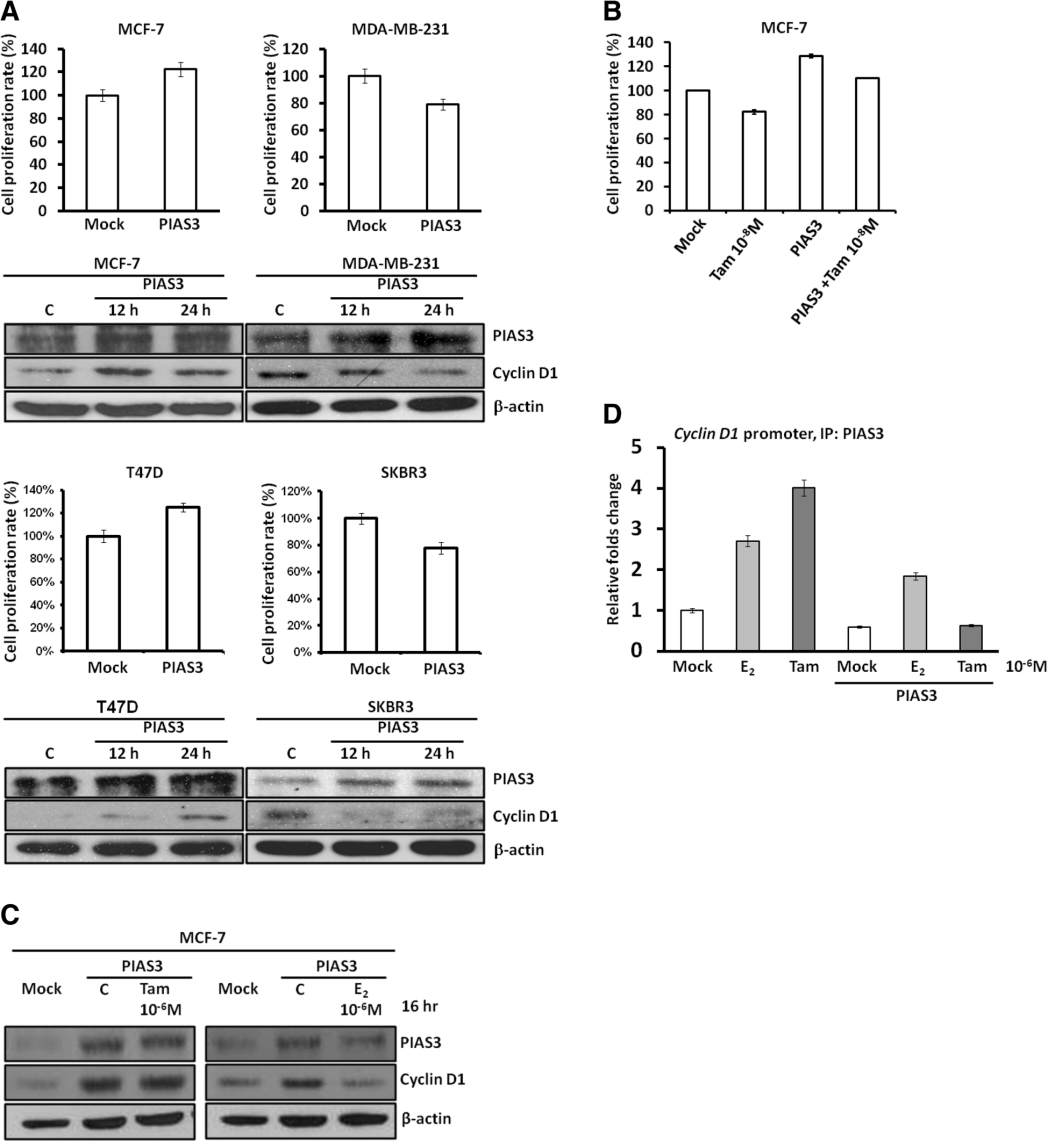
Bioeffects of PIAS3 in breast cancer cells. **a** Ectopic PIAS3 overexpression increased the expression levels of cyclin D1 in estrogen receptor (ER)-positive MCF-7 and T47D cells, but decreased those of cyclin D1 in ER-negative MDA-MB-231 and SKBR3 cells. **b** The XTT results revealed that ectopic PIAS3 overexpression increased proliferation of ER-positive MCF-7 and T47D cells but inhibited proliferation of ER-negative MDA-MB-231 and SKBR3 cells. Ectopic PIAS3 overexpression attenuated the cytotoxicity of Tam to ER-positive MCF-7 cells. **c** Increased cyclin D1 expression upon ectopic PIAS3 overexpression was not reversed by Tam, but was inhibited by 17β-estradiol (E2) in ER-positive MCF-7 cells. **d** Chromatin immunoprecipitation revealed that ectopic PIAS3 overexpression decreased its binding to the conserved STAT3-binding element of the *cyclin D1* promoter. Tam did not reverse the decreased trend of PIAS3 binding to the *cyclin D1* promoter, while E2 increased PIAS3 binding to the *cyclin D1* promoter after ectopic PIAS3 overexpression in ER-positive MCF-7 cells

According to our clinical observations, PIAS3 expression appeared to interrupt the therapeutic effects of hormone therapy mainly based on ER status. To determine the potential roles of PIAS3 in breast cancer regarding ER status, we transiently introduced the PIAS3 expression plasmid into ER-positive MCF-7 and T47D cells as well as ER-negative MDA-MB-231 and SKBR3 cells. Unexpectedly, ectopic PIAS3 overexpression increased proliferation of MCF-7 and T47D cells but inhibited proliferation of MDA-MB-231 and SKBR3 cells (Fig. 3a). Meanwhile, PIAS3 increased cyclin D1 expression in MCF-7 and T47D cells but inhibited the same in MDA-MB-231 and SKBR3 cells (Fig. 3a), suggesting that different effects of PIAS3 on breast cancer cells may be modulated by ER. Because of our clinical observations showed that increased PIAS3 may reduce the overall survival of patients who were mostly ER positive and were receiving Tam-based hormone therapy, we further examined if Tam would change the PIAS3-mediated proliferation and cyclin D expression in ER-positive MCF-7 cells. We found that ectopic PIAS3 overexpression significantly attenuated the cytotoxicity of Tam in ER-positive MCF-7 cells (Fig. 3b). In addition, increased cyclin D1 expression upon ectopic PIAS3 overexpression was not reversed by Tam but was unexpectedly inhibited by 17β-estradiol (E2) (Fig. 3c). It has been clearly demonstrated that PIAS3 can decrease DNA binding of STAT3, as well as of itself, and inhibit the transcription activity of STAT3. Accordingly, ectopic PIAS3 overexpression decreased its binding to the conserved and proven STAT3-binding site on the *cyclin D1* promoter in MCF-7 cells (Fig. 3d). This decreased binding of PIAS3 on the *cyclin D1* promoter may explain why its overexpression would lead to increased cyclin D1 expression. Consistently, Tam did not reverse this binding trend and E2 retained binding of PIAS3 on the *cyclin D1* promoter after PIAS3 overexpression (Fig. 3d).

## Discussion

Aberrant STAT3 signaling has been associated with breast cancer. Therefore, it is important to understand the clinical significance and underlying mechanisms of its downstream, negative feedback regulator, PIAS3, in breast cancer. Our results showed that the total PIAS3 level was mainly lower in the breast cancer tissues than in the adjacent noncancerous tissues, suggesting that a reduction of PIAS3 levels in native breast tissues may be associated with breast cancer development. Consistently, lower PIAS3 expression has also been observed in gastric cancer and squamous cell carcinoma of the lung [20, 21]. Although the correlations did not achieve significance, we found that decreased PIAS3 expression may contribute to advanced breast cancer development including the advanced tumor staging and positive node metastasis observed in our cases (Table 1). Notably, highly metastatic and aggressive ER-negative breast cancer cell lines have relatively lower PIAS3 expression when compared with ER-positive breast cancer cell lines (Additional file 1: Figure S1).

Growing evidence has revealed that ectopic PIAS3 overexpression can inhibit cell proliferation and induction of apoptosis in cancer cells under certain conditions [22, 23]. Our results showed that ectopic PIAS3 overexpression inhibited both proliferation (Fig. 3a) and migration (data not shown) of highly metastatic ER-negative MDA-MB-231 cells. However, PIAS3 overexpression stimulated cell growth of ER-positive MCF-7 and T47D cells. Accordingly, PIAS3 increased the expression of cyclin D1 in MCF-7 and T47D cells, but decreased the same in MDA-MB-231 and SKBR3 cells. This suggests that other co-regulators (i.e., ER) may change the functions and activities of PIAS3 at least in breast cancer.

Induction of *cyclin D1* gene transcription by ER plays an important role in estrogen-mediated proliferation; however, no classical estrogen response element is present in the *cyclin D1* promoter [24, 25]. It has also been demonstrated that estrogen induces the expression of the *PIAS3* gene and increases the physical association between PIAS3 and STAT3 [12]. Unexpectedly, ectopic PIAS3 overexpression increased ligand (e.g., estrogen, E2)-independent cyclin D1 elevation in our study. We also found that ectopic PIAS3 overexpression promoted ligand-independent proliferation of ER-positive breast cancer cells but inhibited cell growth and cyclin D1 expression in ER-negative breast cancer cells. We hypothesize that the difference of PIAS3-mediated effects on cell growth between the two types of cancer cell lines is due to the presence of ER. It has been suggested ER can interact with STAT3 to downregulate STAT3 signaling such as cyclin D1 in our case [12]. We found that ectopic PIAS3 overexpression increased the activation of STAT3 in ER-positive MCF7 cells and decreased the activation of STAT3 in MDA-MB-231 cells (Additional file 2: Figure S2A). Accordingly, we found that several downstream genes of STAT3 were downregulated in MDA-MB-231 cells (Additional file 2: Figure S2B). However, the expression levels of cyclin D1 and c-myc, but not those of Bcl-2 and Mcl-1, were increased upon PIAS3 overexpression in MCF7 cells, suggesting that the PIAS3-mediated effects on ER-positive breast cancer cells may be promoter-specific or ER-related. In the present study, PIAS3 may interrupt or decrease the baseline inhibition of STAT3 signaling by ER in ER-positive breast cancer cells. Accordingly, we observed that cyclin D1 expression and proliferation were increased by PIAS3 in ER-positive MCF-7 and T47D cells. In the absence of ER (e.g., MDA-MB-231 and SKBR3 cells), PIAS3 may inhibit STAT3 signaling directly or indirectly.

Interestingly, we also found that increased cyclin D1 expression after ectopic PIAS3 overexpression was not reversed by Tam but was inhibited by E2. In addition, as seen during clinical observations and in vitro studies, increased PIAS3 may attenuate the therapeutic effects of Tam-based hormone therapy in breast cancer patients as well as the cytotoxicity of Tam to the breast cancer cells. This observation is partially supported by a report that Tam stimulates the growth of cyclin D1-overexpressing breast cancer cells [12]. Furthermore, ectopic overexpression of PIAS3 decreased its binding to the *cyclin D1* promoter, leading to an elevation of cyclin D1. Tam treatment did not reverse this trend of reduced binding, but E2 treatment maintained the binding of PIAS3 on the *cyclin D1* promoter after PIAS3 overexpression. Different conformational changes between E2- and Tam-bound ER may contribute to this effect. Taken together, these results suggest that PIAS3 may be involved in Tam resistance because concomitant expression of ER and PIAS3 may promote cell growth even when cells are treated with Tam. Furthermore, our results revealed that hormone therapy improved overall survival only in patients who presented with decreased or low PIAS3 levels.

## Conclusions

Our results revealed that PIAS3 may be a biomarker for predicting hormone therapy stratification in patients with breast cancer. Constitutively expressed/increased PIAS3 expression may attenuate the effectiveness of Tam-based hormone therapy and thus predict poor overall survival.

## Additional files

Additional file 1: Figure S1. Expression patterns of protein inhibitor of activated signal transducers and activators of transcription 3 (PIAS3) was detected in a panel of breast cancer cell lines. Total PIAS3 was determined by immunobotting in various breast cancer cell lines and one normal breast epithelial cell line. PIAS3 expression levels were normalized to the levels of the corresponding β-actin protein. The Image J software was used to compare the expression levels of total PIAS3. ER: estrogen receptor; HME: human mammary epithelial cells. (PDF 73 kb) Additional file 2: Figure S2. Effects of activated signal transducers and activators of transcription 3 (PIAS3) on STAT3 signaling in breast cancer cells. **(A)** Ectopic PIAS3 overexpression increased expression levels of activated STAT3 (p-STAT3, p-tyr705-STAT3) in estrogen receptor (ER)-positive MCF-7 cells, but decreased those of activated STAT3 in ER-negative MDA-MB-231 cells. **(B)** Ectopic PIAS3 overexpression attenuated the expression levels of STAT3 downstream genes including cyclin D1, c-myc, Bcl-2, and Mcl-1 in ER-positive MDA-MB-231 cells. However, the expression levels of cyclin D1 and c-myc, but those of Bcl-2 and Mcl-1, were up-regulated in ER-positive breast cancer cells. (PDF 160 kb)

## Abbreviations

JAK: Janus-activated kinase
STAT3: Signal transducer and activator of transcription 3
PIAS3: Protein inhibitor of activated STAT3
ER: Estrogen receptor
Her2: Human epidermal growth factor receptor 2
PR: Progesterone receptor
E2: 17β-estradiol
Tam: Tamoxifen
XTT: Sodium 3′-[1-[(phenylamino)-carbony]-3,4-tetrazolium]-bis(4-methoxy-6-nitro)benzene-sulfonic acid hydrate
SDS: Sodium dodecyl sulfate
Chip: Chromatin immuneprecipitation
EDTA: (Ethylenedinitrilo) tetraacetic acid
HEPES: 4-(2-Hydroxyethyl)piperazine-1-ethanesulfonic acid, N-(2-Hydroxyethyl)piperazine-N′-(2-ethanesulfonic acid)
KCl: Potassium chloride
