# Supplementary figures and images for: Prognostic value of protein inhibitor of activated STAT3 in breast cancer patients receiving hormone therapy
Source: BMC Cancer. 2016 Jan 14;16:20. doi: 10.1186/s12885-016-2063-1 (PMC4714466; doi:10.1186/s12885-016-2063-1)

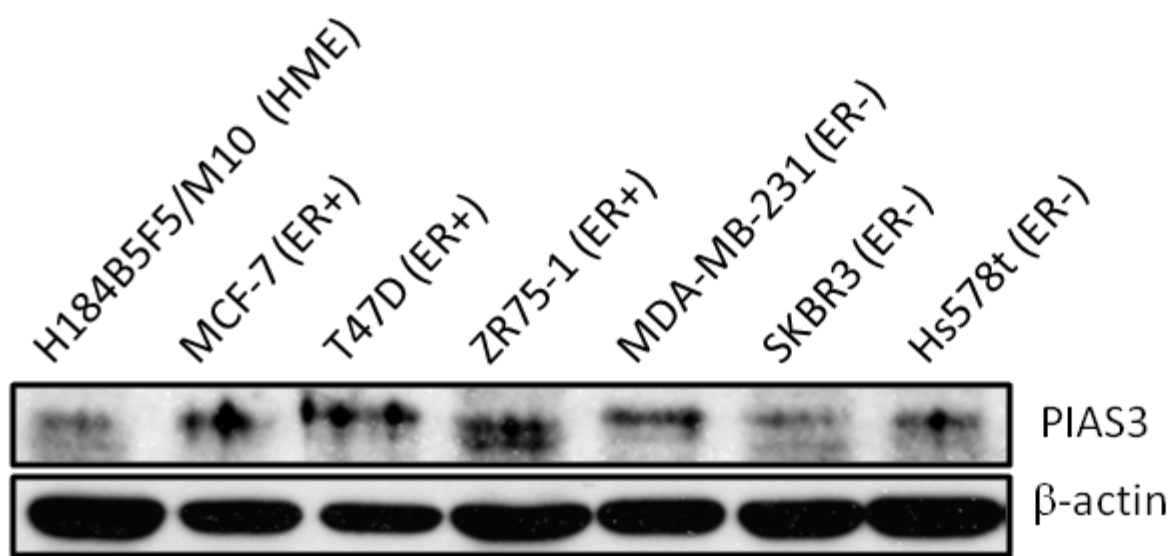

\*Human mammary epithelial cell

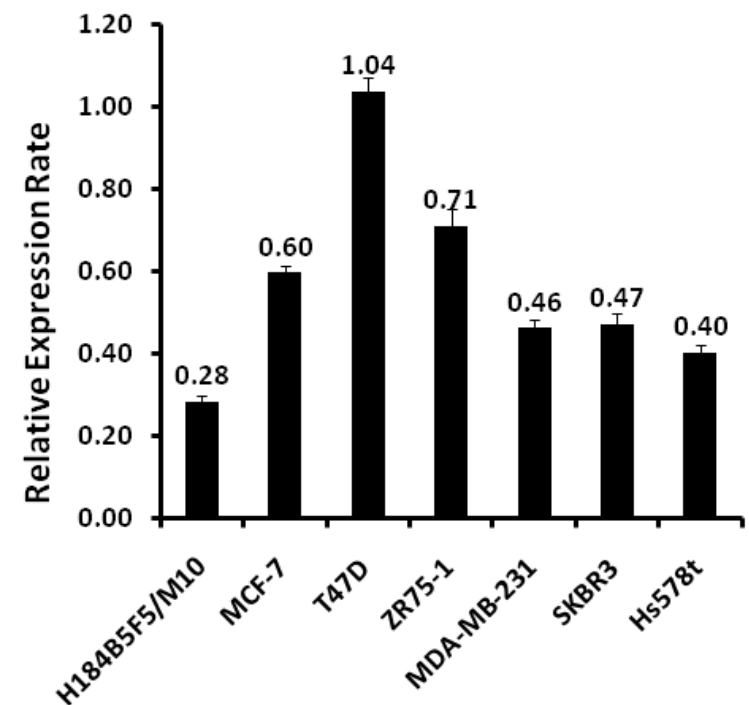

Supplement: Additional file 1: Figure S1. — Expression patterns of protein inhibitor of activated signal transducers and activators of transcription 3 (PIAS3) was detected in a panel of breast cancer cell lines. Total PIAS3 was determined by immunobotting in various breast cancer cell lines and one normal breast epithelial cell line. PIAS3 expression levels were normalized to the levels of the corresponding β-actin protein. The Image J software was used to compare the expression levels of total PIAS3. ER: estrogen receptor; HME: human mammary epithelial cells. (PDF 73 kb) [file 12885_2016_2063_MOESM1_ESM.pdf]

A

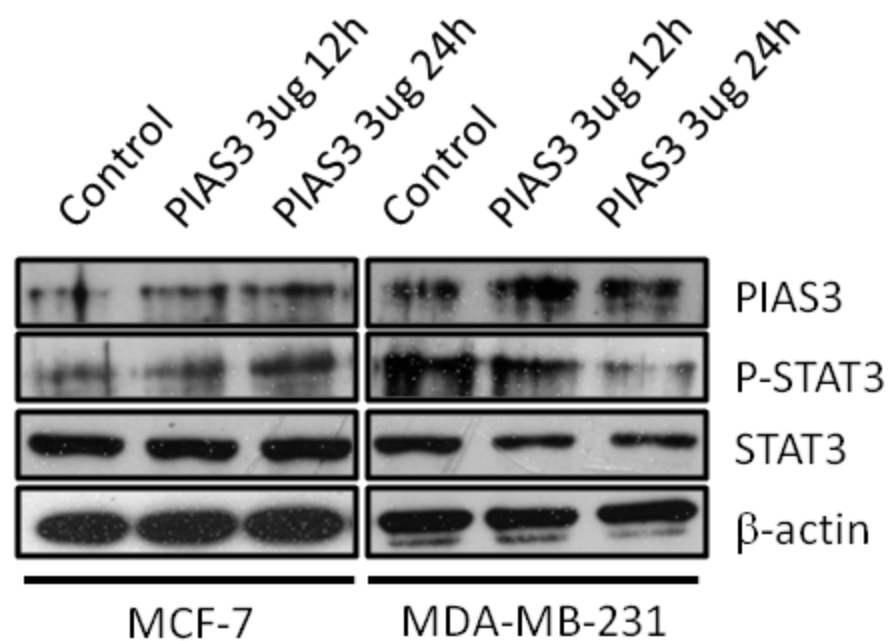

B

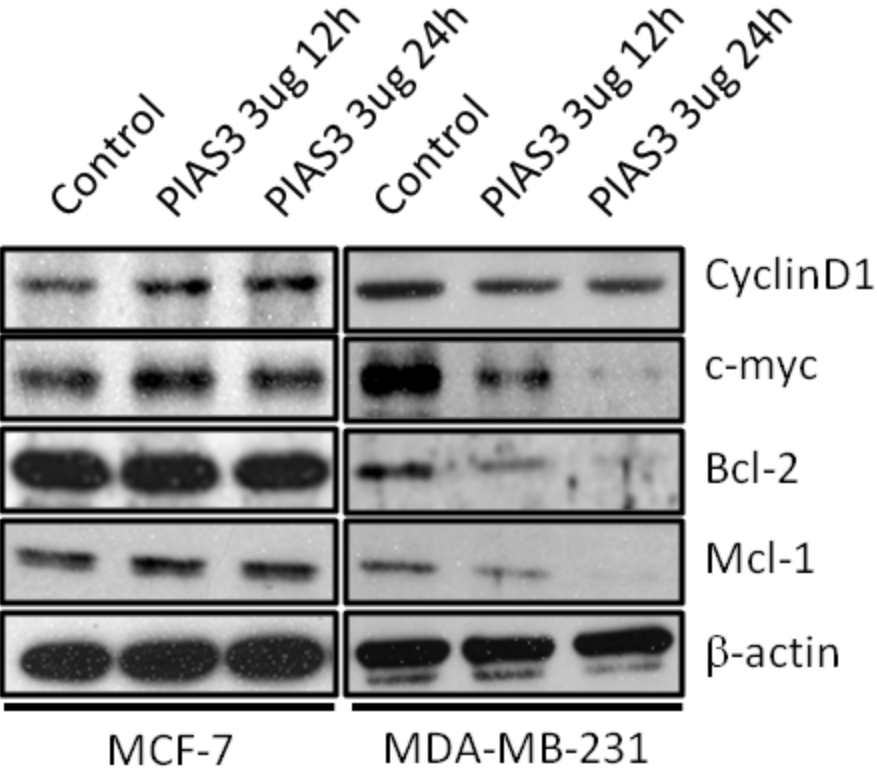

Supplement: Additional file 2: Figure S2. — Effects of activated signal transducers and activators of transcription 3 (PIAS3) on STAT3 signaling in breast cancer cells. (A) Ectopic PIAS3 overexpression increased expression levels of activated STAT3 (p-STAT3, p-tyr705-STAT3) in estrogen receptor (ER)-positive MCF-7 cells, but decreased those of activated STAT3 in ER-negative MDA-MB-231 cells. (B) Ectopic PIAS3 overexpression attenuated the expression levels of STAT3 downstream genes including cyclin D1, c-myc, Bcl-2, and Mcl-1 in ER-positive MDA-MB-231 cells. However, the expression levels of cyclin D1 and c-myc, but those of Bcl-2 and Mcl-1, were up-regulated in ER-positive breast cancer cells. (PDF 160 kb) [file 12885_2016_2063_MOESM2_ESM.pdf]
